# IRS2 silencing increases apoptosis and potentiates the effects of ruxolitinib in JAK2^V617F^-positive myeloproliferative neoplasms

**DOI:** 10.18632/oncotarget.6851

**Published:** 2016-01-09

**Authors:** Paula de Melo Campos, João A. Machado-Neto, Christopher A. Eide, Samantha L. Savage, Renata Scopim-Ribeiro, Adriana da Silva Souza Duarte, Patricia Favaro, Irene Lorand-Metze, Fernando F. Costa, Cristina E. Tognon, Brian J. Druker, Sara T. Olalla Saad, Fabiola Traina

**Affiliations:** ^1^ Hematology and Hemotherapy Center - University of Campinas/Hemocentro - Unicamp, Instituto Nacional de Ciência e Tecnologia do Sangue, Campinas, São Paulo, Brazil; ^2^ Knight Cancer Institute, Oregon Health & Science University, Portland, Oregon, USA; ^3^ Howard Hughes Medical Institute, Portland, Oregon, USA; ^4^ Current address: Department of Biological Sciences, Federal University of São Paulo, Diadema, São Paulo, Brazil; ^5^ Current address: Department of Internal Medicine, University of São Paulo at Ribeirão Preto Medical School, Ribeirão Preto, São Paulo, Brazil

**Keywords:** IRS2, JAK2^V617F^, STAT5, myeloproliferative neoplasms, apoptosis

## Abstract

The recurrent V617F mutation in JAK2 (JAK2^V617F^) has emerged as the primary contributor to the pathogenesis of myeloproliferative neoplasms (MPN). However, the lack of complete response in most patients treated with the JAK1/2 inhibitor, ruxolitinib, indicates the need for identifying pathways that cooperate with JAK2. Activated JAK2 was found to be associated with the insulin receptor substrate 2 (IRS2) in non-hematological cells. We identified JAK2/IRS2 binding in JAK2^V617F^ HEL cells, but not in the JAK2^WT^ U937 cell line. In HEL cells, IRS2 silencing decreased STAT5 phosphorylation, reduced cell viability and increased apoptosis; these effects were enhanced when IRS2 silencing was combined with ruxolitinib. In U937 cells, IRS2 silencing neither reduced cell viability nor induced apoptosis. IRS1/2 pharmacological inhibition in primary MPN samples reduced cell viability in JAK2^V617F^-positive but not JAK2^WT^ specimens; combination with ruxolitinib had additive effects. *IRS2* expression was significantly higher in CD34^+^ cells from essential thrombocythemia patients compared to healthy donors, and in JAK2^V617F^ MPN patients when compared to JAK2^WT^. Our data indicate that IRS2 is a binding partner of JAK2^V617F^ in MPN. IRS2 contributes to increased cell viability and reduced apoptosis in JAK2-mutated cells. Combined pharmacological inhibition of IRS2 and JAK2 may have a potential clinical application in MPN.

## INTRODUCTION

Chronic myeloproliferative neoplasms (MPN) are characterized by clonal defects that result in increased myeloid proliferation with preserved terminal differentiation [1]. The identification of a recurrent mutation in the tyrosine kinase Janus kinase 2 (JAK2^V617F^) in most patients with primary myelofibrosis (PMF), polycythemia vera (PV) and essential thrombocythemia (ET) suggest a potential common main cause of disease development [2, 3]. However, the description of other recurrent mutations, the evidence that MPN patients can respond to JAK2 inhibitors regardless of JAK2 mutation status, and the knowledge that many receptors and substrates may lead to the activation of the JAK/STAT, MAPK and PI3K/AKT/mTOR pathways through mechanisms other than the JAK2 pathway suggest that other interacting proteins may be involved in the pathophysiology of these diseases [4, 5].

In non-hematological cells, previous studies have reported that JAK2 can associate with the insulin receptor substrate 2 (IRS2). JAK2 co-immunoprecipitates with IRS2 following angiotensin II stimulation in the left ventricle of rats [6, 7], and upon leptin stimulation in the rat liver [8]. IRS2 is a 180kDa adapter protein that was described in 1995 as being the equivalent to the 4PS protein, initially identified as a substrate of the IL4 receptor-associated tyrosine kinase in myeloid cells [9]. IRS2 associates with the p85 subunit of PI3K and with GRB2 and mediates mitogenic and antiapoptotic signaling from IR, IGF1R, EPOR, MPL, VEGF, insulin, leptin, GH, interleukins and IFNα/β/γ, which can influence the proliferation of neoplastic cells [7, 9–17]. During the erythroid maturation of normal and PV cells, insulin and IGF1 induce cell signaling through IRS1 and IRS2 phosphorylation [18, 19]. IRS2 expression is higher in aggressive, metastatic human breast carcinoma cell lines [16], and mammary tumor cells that are deficient for IRS2 expression are significantly more sensitive to apoptotic stimuli, such as serum deprivation [16, 20, 21]. We have previously identified IRS2 phosphorylation to be upregulated during hematopoietic cell differentiation [22], although the functional role of IRS2 in hematopoietic neoplasms has not yet been addressed.

We hypothesized that IRS2 could participate in the activation of crucial signaling pathways in MPN via direct binding with JAK2 or through alternative mechanisms. In this study, we aimed to evaluate JAK2/IRS2 association and to describe the function of IRS2 in cell viability and apoptosis using cell lines and primary samples from MPN patients harboring wild-type or mutant JAK2. *IRS2* mRNA expression levels were investigated in primary CD34^+^ cells from healthy donors and patients with MPN; *IRS2* mRNA expression was compared between these groups and among MPN patients stratified according to *JAK2* and *CALR* mutational status.

## RESULTS

### IRS2 is constitutively associated with JAK2 in HEL cells

**Figure 1:**
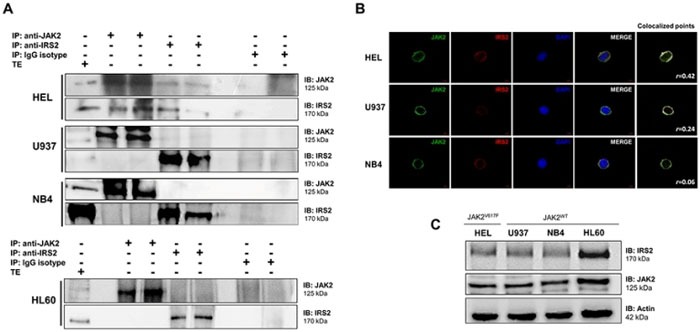
IRS2 associates with JAK2 in HEL cells **A.** Immunoprecipitation (IP) and immunoblotting (IB) with anti-IRS2 and JAK2 antibodies showed a constitutive association between IRS2 and JAK2 in HEL cells harboring the JAK2^V617F^ mutation, but not in JAK2^WT^ cell lines U937, NB4 and HL60. Isotype IgG antibody was used as a negative control of the immunoprecipitation; total cell extracts were used as positive controls for immunoblotting. Blots were cropped to improve the clarity of the figure and retain important bands. **B.** Confocal analysis of HEL, U937 and NB4 cells displaying JAK2 (green), IRS2 (red) and DAPI (blue) staining; MERGE shows the overlapped images. Colocalization analysis was performed with the “colocalization finder” plug-in of Image J NIH software, and shows merged images of JAK2 and IRS2, with colocalized points in white. The correlation coefficient (*r*) values are indicated. **C.** Immunoblotting (IB) with anti-IRS2 and JAK2 antibodies showed high IRS2 and JAK2 expression in HEL, U937, NB4 and HL60 cell lines.

Leukemia cell lines harboring the JAK2^V617F^ mutation (HEL) or JAK2^WT^ (U937, NB4, HL60) were used for immunoprecipitation and immunoblotting with anti-IRS2 and anti-JAK2 antibodies. Immunoprecipitation analysis revealed that JAK2 binds to IRS2 in HEL JAK2^V617F^ cells, but not in U937, NB4 and HL60 JAK2^WT^ cell lines (Figure 1A). Similarly, colocalization of IRS2 and JAK2 by confocal microscopy was higher in HEL cells in comparison to U937 and NB4 cells (Figure 1B; Supplementary Figure S1). JAK2 and IRS2 protein expressions in these cell lines are illustrated in Figure 1C.

### Ruxolitinib decreases IRS2 phosphorylation in JAK2^V617F^ cells

**Figure 2:**
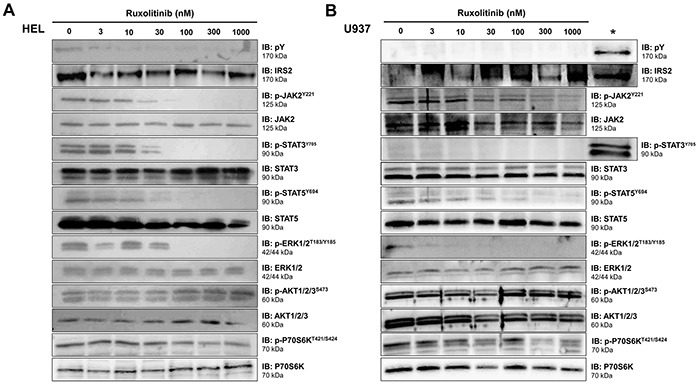
Ruxolitinib decreases IRS2 phosphorylation in JAK2^V617F^ cells Total cell extracts of **A.** JAK2^V617F^ HEL cells or **B.** JAK2^WT^ U937 cells treated with different doses of ruxolitinib for 6h were submitted to immunoblotting (IB) analysis with anti-IRS2, anti-phosphotyrosine, and antibodies to detect downstream proteins. The asterisk (*) indicates HEL cells used as a positive control along with U937 cells. Membranes were re-probed with the antibody for detection of the respective total and phospho-protein, and developed with the ECL^TM^ Western Blot Analysis System. Blots were cropped to improve the clarity of the figure and retain important bands.

To verify the effects of ruxolitinib treatment on IRS2 phosphorylation and downstream pathways in cells expressing wild-type or mutant JAK2, HEL and U937 cells treated with increasing concentrations of ruxolitinib for 6h were tested for total and phospho-proteins by immunoblotting with specific antibodies. In HEL cells, ruxolitinib treatment resulted in decreased phosphorylation of IRS2, JAK2, STAT3, STAT5, ERK and P70S6K, but no changes were observed in phospho-AKT levels (Figure 2A). In contrast, U937 cells showed little to no phosphorylated IRS2 and STAT3 even in the absence of ruxolitinib treatment, but demonstrated reduced JAK2, STAT5, ERK and P70S6K phosphorylation upon ruxolitinib administration (Figure 2B). NB4 cells, which also harbor wild-type JAK2, showed neither constitutive phosphorylation of JAK2 nor any reduction in downstream signaling proteins upon ruxolitinib treatment (Supplementary Figure S2). Given these findings, HEL and U937 cells were selected for further study as models of JAK2^V617F^ and JAK2^WT^ signaling.

### IRS2 silencing decreases STAT5 phosphorylation in HEL cells

**Figure 3:**
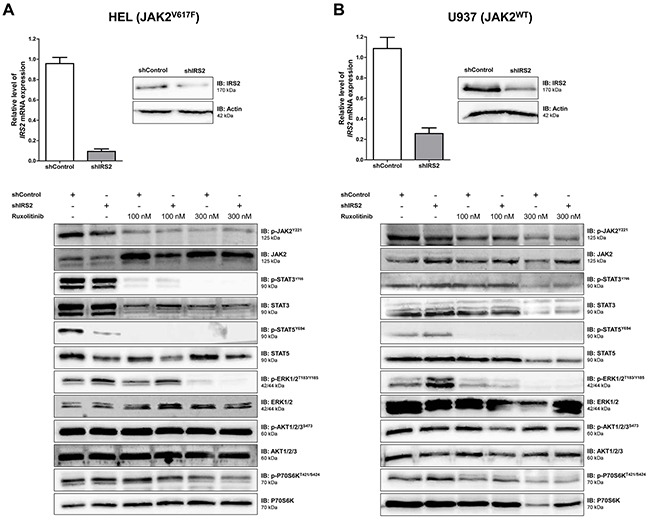
IRS2 silencing decreases STAT5 phosphorylation in HEL (JAK2^V617F^) cells, but not in U937 (JAK2^WT^) cells **A.** HEL cells or **B.** U937 cells were transduced with lentivirus-mediated shRNA control (shControl) or IRS2 (shIRS2). IRS2 mRNA and protein expression in shIRS2 cells relative to the shControl cells (upper panel). Western blot analysis for total and phospho-proteins JAK2, STAT3, STAT5, ERK, AKT and P70S6K in total cell extracts of shControl and shIRS2 HEL or U937 cells treated with ruxolitinib (100 or 300nM) or DMSO for 48h (lower panel). The antibodies used for immunoblotting (IB) are indicated; membranes were reprobed with the antibody for detection of the respective total and phospho-protein or actin, and developed with the ECL Western Blot Analysis System. Blots were cropped to improve the clarity of the figure and retain important bands.

To investigate the function of IRS2 in wild-type and mutant JAK2 cells, HEL and U937 cells were efficiently silenced for IRS2 through stable transduction with lentiviral constructs encoding shRNA targeting *IRS2* (shIRS2) or a shRNA targeting a non-specific control sequence (shControl), as verified by qPCR and western blotting (Figure 3A–3B). To determine the combined effects of IRS2 inhibition and ruxolitinib treatment on JAK/STAT, PI3K/AKT/mTOR and MAPK signaling, shControl and shIRS2 cells were treated with DMSO or ruxolitinib (100 or 300nM) for 48h, and submitted to immunoblotting with specific antibodies. In HEL cells, IRS2 silencing alone was able to induce decreased phosphorylation of STAT5 and increased phospho-ERK levels. Ruxolitinib downregulated JAK/STAT (decreased phosphorylation of JAK2, STAT3 and STAT5) and MAPK signaling (decreased phosphorylation of ERK and P70S6K), but did not modulate AKT phosphorylation in HEL cells (Figure 3A). In JAK2^WT^ U937 cells, however, while IRS2 silencing did not change STAT5 phosphorylation, increased phospho-ERK levels were observed (Figure 3B). The individual effects of IRS2 silencing were not observed in cells submitted to ruxolitinib 300nM treatment, since such treatment results in near complete inhibition of phospho-STAT5 and phospho-ERK by 48h of exposure (Figure 3A–3B).

### IRS2 silencing decreases cell viability and potentiates the effect of ruxolitinib in JAK2^V617F^ cells

**Figure 4:**
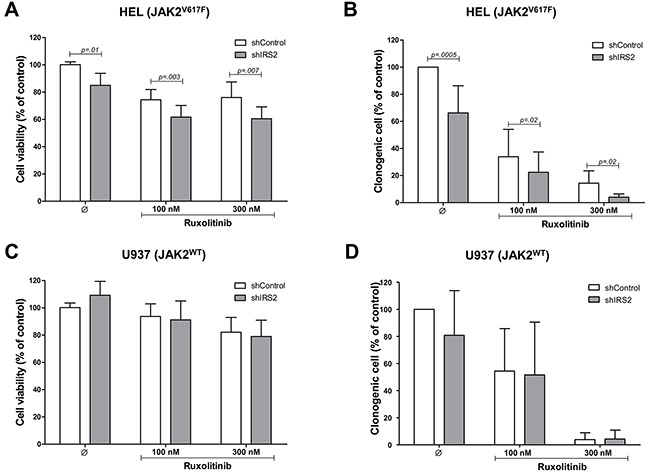
IRS2 silencing reduces cell viability and colony formation, and potentiates the effects of ruxolitinib in HEL cells **A.** Cell viability was determined by methylthiazole tetrazolium (MTT) assay after 48h of incubation of shIRS2 HEL cells and normalized by the corresponding shControl HEL cells. Results are shown as mean ± SD of eight independent experiments; Mann–Whitney test. **B.** Colonies containing viable cells were detected by MTT after 8 days of culture of shIRS2 HEL cells and normalized by the corresponding shControl HEL cells. The percentage of colonies relative to controls are shown in the bar graph as mean ± SD of twelve independent experiments; Student's t-test. The assays were performed in the presence of ruxolitinib (100 or 300mM) or DMSO, as indicated. **C.** U937 cells stably-transduced with lentivirus-mediated shRNA control (shControl) or IRS2 (shIRS2) were submitted to MTT assay or **D.** colony forming assay, as indicated. All conditions were tested in at least eight independent experiments, and no statistically significant difference was observed for U937 cells.

To evaluate the role of IRS2 on cell viability and clonogenicity, cells were silenced for IRS2 and submitted to MTT (methylthiazole tetrazolium) or colony formation assays, respectively. To assess the combined effects of IRS2 knockdown and JAK1/2 inhibition, shIRS2 or shControl cells were treated with ruxolitinib (100 and 300nM) or DMSO for 48h (cell viability) or 8 days (colony formation). HEL cell viability and clonogenicity were significantly inhibited by IRS2 silencing (*p* ≤.01), and these effects were enhanced when IRS2 silencing was combined with ruxolitinib exposure (all *p* ≤.02) (Figure 4A–4B). In U937 cells, IRS2 silencing had no effects on cell viability and clonogenicity; ruxolitinib did not significantly suppress cell viability, but decreased the clonogenicity of these cells (Figure 4C–4D), likely due to the time of drug exposure necessary for each assay.

### IRS2 silencing induces apoptosis and potentiates the effects of ruxolitinib in HEL cells

**Figure 5:**
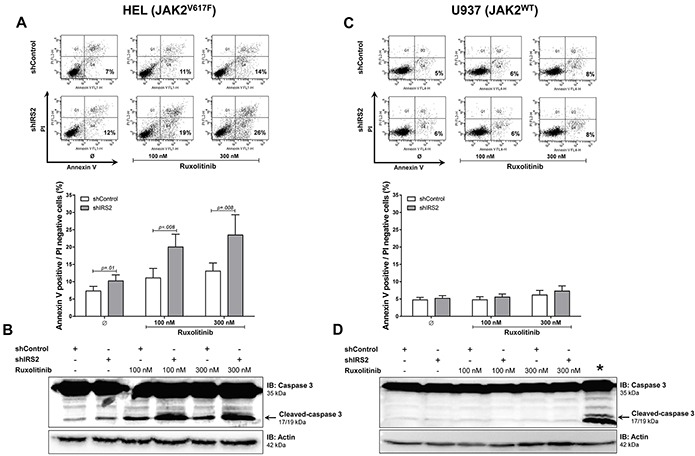
IRS2 silencing induces apoptosis and has cumulative effects with ruxolitinib in HEL cells **A.** Apoptosis was detected by flow cytometry in HEL cells transduced with shControl and shIRS2 using Annexin V^+^/PI^−^ staining method and a representative dot plot is illustrated. Bar graphs are shown as mean ± SD of eight independent experiments; Student's t-test. The assays were performed in the presence of ruxolitinib (100 and 300mM) or DMSO for 48h, as indicated. **B.** All conditions were submitted to imunoblotting (IB) analysis for cleaved caspase 3, and a representative experiment is shown. Membranes were reprobed with the antibody for detection of actin, and developed with the ECL Western Blot Analysis System. Blots were cropped to improve the clarity of the figure and retain important bands. **C.** shControl or shIRS2 U937 cells treated with DMSO only or ruxolitinib (100 or 300nM) for 48h were submitted to Annexin V/PI staining and **D.** imunoblotting for cleaved caspase 3, as indicated. All conditions were tested in at least eight independent experiments, and no statistically significant difference in apoptosis was observed for U937 cells; no cleaved caspase 3 was observed in any condition. The asterisk (*) indicates ruxolitinib-treated HEL cells used as a positive control for apoptosis along with U937 cells.

IRS2 silencing significantly induced apoptosis and potentiated the effects of ruxolitinib treatment in HEL cells (all *p* ≤.01) (Figure 5A). Consistent with this, increased caspase 3 cleavage was observed by immunoblotting in shIRS2 cells, with greater levels observed upon additional ruxolitinib treatment (Figure 5B). However, in U937 cells, IRS2 silencing alone or associated with ruxolitinib did not affect apoptosis, as assessed by Annexin V^+^/PI^−^ and immunoblotting with anti-cleaved caspase 3 antibodies (all *p* ≥.05). Of note, ruxolitinib did not induce apoptosis in U937 cells (Figure 5C–5D).

### Increased *IRS2* mRNA expression in patients harboring the JAK2^V617F^ mutation

**Figure 6:**
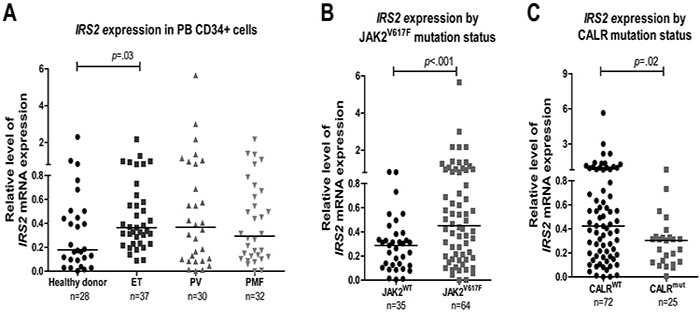
Increased *IRS2* mRNA levels in CD34^+^ cells from JAK2^V617F^-positive myeloproliferative neoplasm patients **A.** qPCR analysis of *IRS2* mRNA expression in peripheral blood (PB) CD34^+^ cells from healthy donors and patients with the diagnosis of essential thrombocythemia (ET), polycythemia vera (PV) and primary myelofibrosis (PMF), and stratified by **B.** JAK2^V617F^ or **C.** CALR exon 9 mutational status. Horizontal lines indicate medians. The number of subjects and the *p* values (Mann–Whitney test) are indicated.

*IRS2* mRNA levels were evaluated in peripheral blood CD34^+^ cells from healthy donors and patients with MPN. Significantly increased *IRS2* mRNA expression was observed in patients with ET when compared to healthy donors (0.35 [0.08-2.16] *versus* 0.18[0.00-2.29]; *p* =.03). A slight, although not statistically significant, increase in *IRS2* mRNA expression was found in PV (0.36 [0.00-5.65]) and PMF (0.31 [0.00-2.16]) patients when compared to healthy donors (Figure 6A). Significantly higher levels of *IRS2* mRNA expression were also observed in JAK2^V617F^ patients when compared to JAK2^WT^ (0.46 [0.00-5.65] *versus* 0.29 [0.00-0.83]; *p*<.001) (Figure 6B). This difference remained significant when comparing JAK2^V617F^ and JAK2^WT^ patients in individual groups (ET, PMF) (*p* ≤.02). Despite the low *IRS2* mRNA expression in JAK2^WT^ PV patients, the number of wild-type samples was excessively small (n=2) for statistical analysis (Supplementary Table S3). *IRS2* mRNA expression was significantly lower in CALR^exon9indel_MUT^ patients compared to CALR^WT^ (0.30 [0.00-0.82] *versus* 0.42 [0.00-5.65]; *p*=.02) (Figure 6C); this difference in *IRS2* expression would be expected since, in this cohort, JAK2^V617F^ and CALR mutations were mutually exclusive, and CALR^MUT^ patients represent the JAK2^WT^ patients.

### IRS2 inhibition decreases cell viability in JAK2^V617F^ primary cells and potentiates the efficacy of ruxolitinib

**Figure 7:**
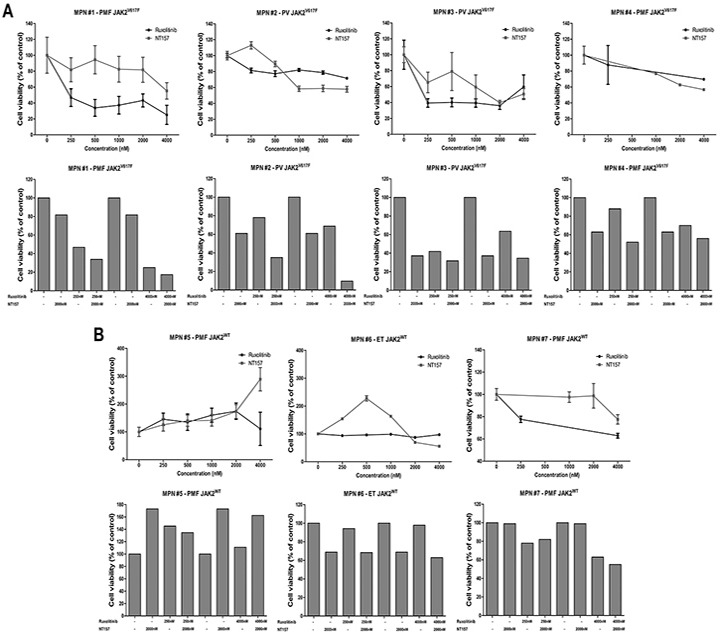
IRS2 pharmacological inhibition decreases cell viability in JAK2^V617F^ myeloproliferative neoplasm primary cells and has cumulative effects with ruxolitinib Cell viability was determined by methanethiosulfonate (MTS)-based assay (patients #1 to #3, #5 and #6) or by methylthiazole tetrazolium (MTT) (patients #4 and #7) after 72h of incubation of JAK2^V617F^ **A.** or JAK^WT^ **B.** myeloproliferative neoplasms (MPN) patients' peripheral blood mononuclear cells with different concentrations of NT157 (IRS1/2 inhibitor) or ruxolitinib (JAK1/2 inhibitor) alone or in combination, as indicated. All conditions were tested in triplicate. Data were normalized to the mean percent of the untreated control wells. PMF: primary myelofibrosis; PV: polycythemia vera; ET: essential thrombocythemia.

To evaluate the combined effects of IRS2 and JAK1/2 pharmacological inhibition in MPN primary cells, mononuclear cells isolated from peripheral blood samples obtained from four JAK2^V617F^ and three JAK2^WT^ MPN patients were submitted to *ex vivo* treatment with the IRS/IGF1R inhibitor NT157, ruxolitinib, or both drugs combined. Six of the seven patients included in the *ex vivo* experiments were *MPL* and *CALR* wild type; one patient with ET (sample #6) who had wild type *JAK2* and *CALR* harbored a *MPL*^W515L^ mutation. In JAK2^V617F^-positive primary cells from PMF and PV patients (samples #1 to #4), a reduced cell viability following NT157 and ruxolitinib exposure alone was observed, and the combined treatment with both inhibitors resulted in cumulative effects (Figures 7A). In JAK2^WT^ primary cells (samples #5 to #7), NT157 reduced cell viability only in sample #6 (ET patient, *MPL*^W515L^-mutated), while ruxolitinib reduced cell viability only in sample #7 (PMF patient). Of note, no combined effect of NT157 plus ruxolitinib was noted in JAK2^WT^ samples (Figures 7B).

## DISCUSSION

IRS2 mediates downstream signaling from EPOR and MPL in hematopoietic cells [14, 23] and associates with JAK2 upon an extrinsic stimulus in non-hematological cells [6–8]. We hypothesized that the constitutive activation of JAK2 induced by the V617F mutation itself could lead to JAK2/IRS2 binding, and that this protein association could participate in the malignant phenotype of JAK2^V617F^-positive MPN patients. We herein describe that IRS2 is a binding partner of JAK2 in the human JAK2^V617F^-positive HEL cell line, an association which was not observed in U937, NB4 and HL60 cells (JAK2^WT^). The lack of the JAK2/IRS2 association in U937, NB4 and HL60 cells corroborates the hypothesis that activated JAK2 is crucial for IRS2 recruitment.

Erythropoeitin induces IRS2 upregulation in primary erythroblasts and in human acute myeloid leukemia cells UT7 [14, 24]. Interestingly, IRS2 can associate directly with both EPOR [14] and JAK2 [6]. JAK2 tyrosine phosphorylation by EPOR or by other mechanisms leads to the recruitment of SH2 and PTB domain-containing proteins and initiates major tyrosine phosphorylation [25, 26]. As such, the IRS2 PTB domain may be involved in the JAK2/IRS2 association in JAK2^V617F^ cells. Alternatively, Duan *et al.* [27] demonstrated that the SH2B adaptor protein binds simultaneously to both JAK2 and IRS2, promoting the formation of a JAK2/SH2B/IRS2 tertiary complex and the subsequent tyrosine phosphorylation of IRS2 by JAK2. Leptin has been shown to induce JAK2/IRS2 association in HEK293 cells expressing leptin receptor only in the presence of SH2B, and leptin was able to induce neither JAK2 nor IRS2 activation in SH2B^−/−^ mice [28], indicating that an adaptor protein may also participate in the JAK2/IRS2 binding.

We herein demonstrated that IRS2 phosphorylation is increased in HEL cells, when compared to U937 cells. *IRS2* gene expression is also higher in CD34^+^ cells from patients with ET, when compared to healthy donors, and from patients with JAK2^V617F^ MPN compared to JAK2^WT^ patients, indicating that the JAK2^V617F^ mutation is associated with increased *IRS2* expression. IRS2 lentivirus-mediated silencing reduced cell viability and clonogenicity and increased apoptosis in HEL cells (JAK2^V617F^), but not in U937 cells (JAK2^WT^). This might be explained by the significantly diminished STAT5 tyrosine phosphorylation seen in IRS2-silenced HEL cells. Although phospho-STAT5 was reduced by the JAK1/2 specific inhibitor ruxolitinib, IRS2 silencing alone significantly downregulated STAT5 activation, suggesting that, in HEL cells, STAT5 may be activated both by an IRS2-dependent/JAK2-independent and JAK2-dependent mechanism. This could explain the additive effects observed in IRS2-silenced/ ruxolitinib-treated cells. Although STAT5 is a classical downstream protein activated by JAK2, insulin can also induce STAT5 activation in a JAK2-independent manner [29]. In the rhabdomyosarcoma cell line Kym-1, an insulin receptor kinase inhibitor abolished the insulin-induced STAT5 tyrosine phosphorylation, but this effect was not obtained with a JAK2 kinase inhibitor [29]. Recently, Schafranek and colleagues have also shown that the loss of STAT5 activity, rather than JAK2, is critical for the irreversible induction of cell death in BCR-ABL-positive cells [30]. Since STAT5 activation decreases apoptosis [31–34] and induces malignant transformation and tumor progression [32, 35–38], the STAT5 downregulation promoted by IRS2 inhibition may contribute to the reduced tumor proliferation and survival in HEL cells. Interestingly, IRS2 silencing resulted in increased phospho-ERK expression in both HEL and U937 cells, although IRS2 inhibition reduced survival only in HEL cells. These data suggest that the increased ERK phosphorylation induced by IRS2 silencing itself is not enough to induce the observed effects on cell survival. The mechanism of ERK phosphorylation and the biological consequence of this finding needs to be better clarified in future studies, although, at least in the cell models tested here, the biological effects of IRS2 inhibition seem to be independent of ERK phosphorylation status.

The insulin receptor substrates, as major mediators of downstream pathways involved in cell proliferation and survival, are important proteins for cancer pathogenesis [11, 16, 20, 21, 39, 40]. Pharmacological IRS inhibition was able to inhibit colony formation and cell migration of human melanoma [39] and prostate cancer cells [41]. Interestingly, however, no effects on the survival of normal melanocytes were observed [39]. Mammary tumor cells deficient for IRS2 expression are more sensitive to apoptotic stimuli [20]. We herein showed that the *ex vivo* treatment of JAK2^V617F^ MPN primary cells with the IRS/IGF1R inhibitor NT157 resulted in reduced cell viability and had cumulative effects with ruxolitinib. Interestingly, no evident modulation of cell viability was observed in JAK2^WT^ primary cells following NT157 or ruxolitinib exposure alone or in combination, corroborating our findings that the constitutive activation of signaling pathways driven by JAK2^V617F^, including IRS2/JAK2 binding, is necessary for the biological effects of IRS and JAK1/2 inhibitors. Taken together, these data support the hypothesis that IRS2 contributes to the malignant phenotype of JAK2^V617F^ MPN.

Our data indicate that IRS2 is a binding partner of JAK2^V617F^ and promotes cell survival in MPN. The additive effects of IRS2 silencing in inducing apoptosis observed upon co-treatment with ruxolitinib highlights the potential for use of IRS2 inhibitors in combination with JAK2 inhibitors in the treatment of these diseases. Future research using IRS2 inhibition in *ex vivo* studies, including a larger cohort of MPN patients, and in MPN animal models may help to predict the *in vivo* effectiveness and collateral effects of IRS2 inhibitors.

## MATERIALS AND METHODS

### Cell culture, ruxolitinib treatment and IRS2 silencing

Human cell lines mutated for JAK2^V617F^ (HEL) or wild-type for JAK2 (U937, NB4 and HL60) were used. HEL, U937 and HL60 cells were obtained from ATCC (Philadelphia, PA, USA), NB4 cells were kindly provided by Dr. Eduardo M. Rego (University of São Paulo, Brazil). Cell lines were tested and authenticated by STR matching analysis using the PowerPlex^®^ 16 HS system (Promega, Madison, WI, USA) and the ABI 3500 Sequence Detector System (Applied Biosystems, Foster City, CA, USA); cells were tested in our laboratory before manuscript submission. Cells (2 × 10^5^/mL) were incubated with ruxolitinib (Novartis Pharmaceuticals, Switzerland) or with DMSO for 6 or 48h.

### Transduction of lentivirus

HEL and U937 cells were transduced with lentivirus-mediated shRNA nonspecific control (SC-108080) or lentivirus-mediated shRNA targeting *IRS2* (SC-29378-V) from Santa Cruz Biotechnology (Santa Cruz Biotechnology, Santa Cruz, CA, USA) and named shControl and shIRS2 cells, respectively. Briefly, 2×10^5^ cells were transduced with lentiviral by spinoculation at multiplicity of infection equal to 4.0 and selected by puromycin (0.75μg/mL for HEL and 1.0μg/mL for U937 cells). On day 21, cells were tested for *IRS2* inhibition by qPCR and immunoblotting.

### Real-time quantitative PCR analysis

Cells were submitted to RNA extraction and assayed with cDNA (RevertAid H Minus First Strand cDNA Synthesis Kit; MBI Fermentas, St. Leon-Rot, Germany), SYBR Green Master Mix PCR (MBI Fermentas) and specific primers for *IRS2* and *HPRT* (Supplementary Table S1). Quantitative PCR (qPCR) was performed with an ABI 7500 Sequence Detector System (Applied Biosystems). The relative gene expression was calculated using the equation, 2^−ΔΔCT^.

### Immunoprecipitation and immunoblotting

Equal amounts of protein were used for total extracts or for immunoprecipitation with specific antibodies, followed by SDS-PAGE and Western blot analysis with the indicated antibodies (Supplementary Table S2) and ECL™ Western Blotting Analysis System (Amersham Pharmacia Biotech, UK Ltd., Buckinghamshire, UK). Blots were stripped and reprobed as necessary.

### Confocal immunofluorescence microscopy

HEL, U937, and NB4 cells were grown on cover slips coated with poly-L-lysine (1mg/mL), fixed with 4% paraformaldehyde, permeabilized with 0.5% Triton X-PBS and blocked with 3% bovine serum albumin (BSA) PBS. Cells were then incubated overnight at 4°C with anti-IRS2 or anti-JAK2 antibodies (1:200 in 1% BSA PBS), followed by incubation with secondary antibody (1:400 in 1% BSA PBS) for 2h at room temperature. All incubations were followed by three 5min PBS washes. The slides were mounted in ProLong Gold Anti-Fade Mounting Medium with DAPI (Life Technologies, Carlsbad, USA). Images were generated using a confocal laser-scanning microscope (LSM 510, Carl Zeiss, Welwyn Garden City, UK). The “colocalization finder” plug in of Image J quantification software (U.S. National Institutes of Health, Bethesda, USA) was used for the analysis of JAK2 and IRS2 colocalization analysis.

### Cell viability and colony forming assays

Cell viability was measured by methylthiazole tetrazolium (MTT) assay for HEL and U937 cells. A total of 5×10^4^ ShControl or shIRS2 cells per well were plated in a 96-well plate in RPMI 10% FBS at different concentrations of ruxolitinib (100 or 300nM) or with DMSO for 48h. Briefly, 10μL of a 5mg/mL solution of MTT was added to the wells and incubated at 37°C for 4h. The reaction was stopped by using 100μL of 0.1 N HCl in anhydrous isopropanol and was evaluated by measuring the absorbance at 570nm using an automated plate reader. Colony formation was carried out in semisolid methyl cellulose medium (0.5×10^3^ cell/mL; MethoCult 4230; StemCell Technologies Inc., Vancouver, BC, Canada). Colonies were detected after 8 days of culture by adding 1mg/mL of MTT reagent and scored by Image J quantification software (US National Institutes of Health, Bethesda, MD, USA). For HEL and U937 cell lines, shControl and shIRS2 cells were submitted to colony formation assay in the presence of ruxolitinib in two different concentrations (100 or 300nM) or DMSO for 8 days.

### Assessment of apoptosis

HEL and U937 cells silenced for IRS2 and control cells were seeded on 12-well plates and treated with different concentrations of ruxolitinib (100 or 300nM) or DMSO for 48h. Cells were washed twice with ice cold PBS and resuspended in binding buffer containing 1μg/mL PI and 1μg/mL FITC- or APC- labeled Annexin-V. All specimens were analyzed by flow cytometry using a FACSCalibur (Becton Dickinson, San Jose, CA, USA) after incubation for 15min at room temperature in a light-protected area. Ten thousand events were acquired for each sample. Cleaved-caspase 3 was evaluated by immunoblotting.

### Primary patient samples

Peripheral blood (PB) samples were collected from 99 MPN patients from the outpatient clinic of the University of Campinas (median age 66, range 20-92; PV=30, ET=37, PMF=32) and from 28 healthy donors (median age 44, range 37-51) from the Blood Bank of the institution. Diagnoses were performed according to the 2008 World Health Organization criteria [1]. Among the patients, 64 were positive for the JAK2^V617F^ mutation, 25 had a CALR exon 9 indel mutation, 10 were negative for both mutations, and 2 patients had a JAK2^V617F^ mutation but had not been tested for CALR mutations (due to lack of sample availability). Seventy-one patients were negative for W515L/K *MPL* mutations; twenty-eight patients were not screened for *MPL* mutations due to the unavailability of good quality DNA samples; from those, only five cases were wild type for both *JAK2* and *CALR*. This research was approved by the Institutional and National Review Board in accordance to the Helsinki Declaration; written informed consent was obtained from all subjects. Seventy-two out of 99 patients were in regular use of hydroxyurea at the time of sampling; none had received additional chemotherapy or a JAK1/2 inhibitor before sampling. CD34^+^ cells were isolated from PB mononuclear cells by MIDI-MACS immunoaffinity columns (Miltenyi Biotec, USA) and >90% purity was verified by flow cytometry.

### *Ex vivo* drug combination treatment of primary patient cells

Patients were seen at the Oregon Health & Science University Center for Hematologic Malignancies (patients #1 to #3, #5 and #6) or at the Hematology and Hemotherapy Center of the University of Campinas (patients #4 and #7). Following informed consent, peripheral blood was collected and mononuclear cells were isolated by Ficoll-gradient centrifugation. Cells were resuspended in RPMI 1640 medium supplemented with 10% FBS, L-glutamine, and penicillin/streptomycin and plated in 384-well plates (9000 cells/well) in triplicate in the presence of the JAK1/2 inhibitor ruxolitinib (0-4000nM), the IRS/IGF1R inhibitor NT157 (0-4000nM; Axon MedChem, USA), or both inhibitors in combination at the indicated concentrations. Plates were cultured at 37°C for 72h, and absorbance was measured by a methanethiosulfonate (MTS)-based assay, according to the manufacturer's protocol (CellTiter 96 AQueous One; Promega) for patients #1 to #3, #5 and #6, or by methylthiazole tetrazolium (MTT) assay for patients #4 and #7. Data were normalized to the mean percent of the untreated control wells.

### Statistical analysis

Statistical analyses were performed using GraphPad Instat 5 (GraphPad Software, USA). Shapiro–Wilk was used as a test of normality. For comparisons, an appropriate Student's t-test or Mann–Whitney test was performed. A *p* value <.05 was considered as statistically significant.

## SUPPLEMENTARY FIGURES AND TABLES


