# Supplementary material for: IRS2 silencing increases apoptosis and potentiates the effects of ruxolitinib in JAK2V617F-positive myeloproliferative neoplasms
Source: Oncotarget. 2016 Jan 9;7(6):6948–59. doi: 10.18632/oncotarget.6851 (PMC4872760; doi:10.18632/oncotarget.6851)
Supplement: Supplementary file 1 [file oncotarget-07-6948-s001.pdf]

SUPPLEMENTARY FIGURES AND TABLES

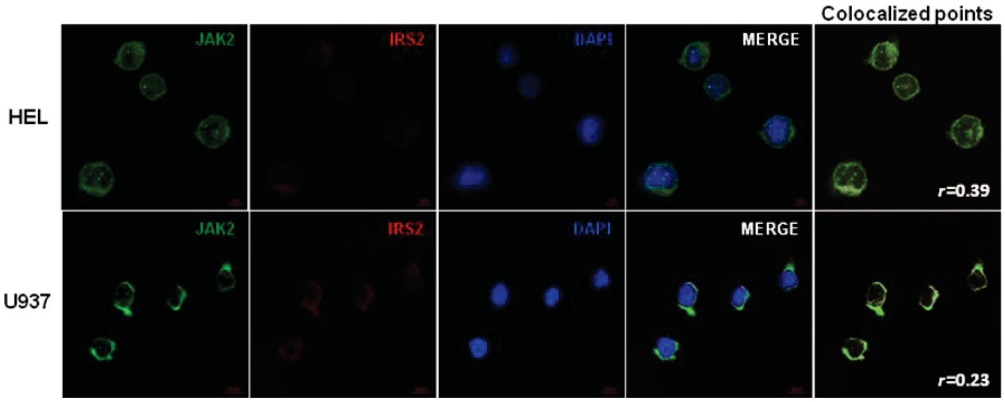

Supplementary Figure S1: Colocalization of IRS2 and JAK2 in HEL cells. Confocal analysis of HEL and U937 cells displaying JAK2 (green), IRS2 (red) and DAPI (blue) staining; MERGE shows the overlapped images. Colocalization analysis was performed with the "colocalization finder" plug-in of the Image J NIH software, and shows merged images of JAK2 and IRS2, with colocalized points in white. The correlation coefficient (r) values are indicated.

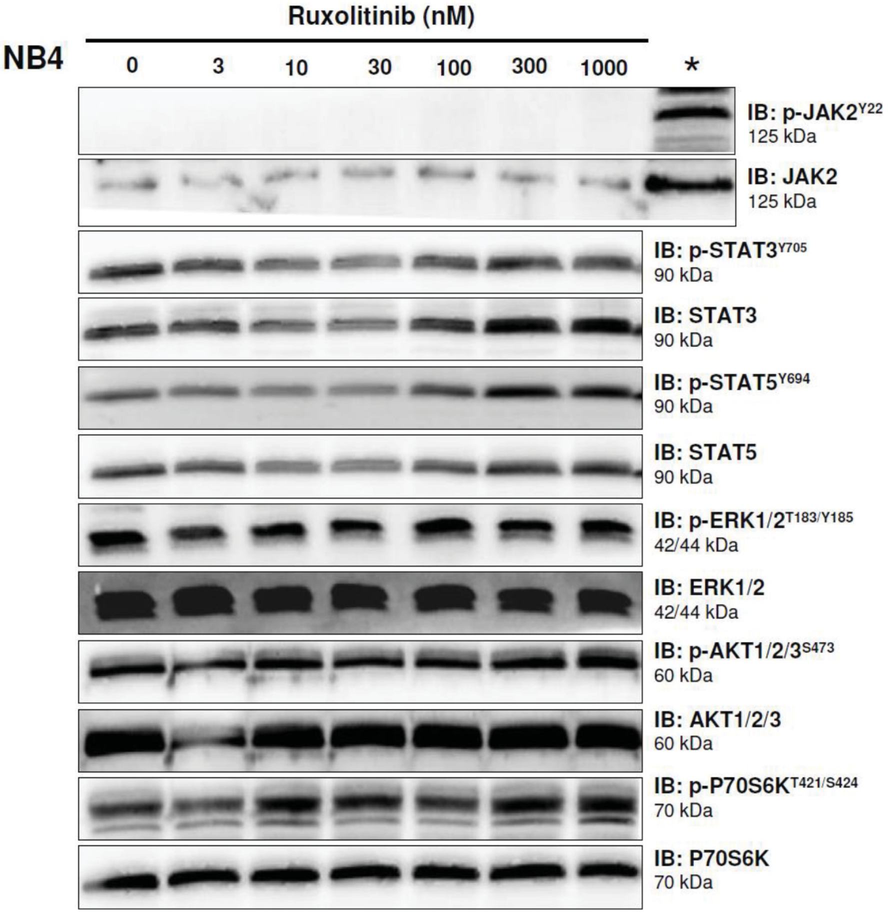

Supplementary Figure S2: Effects of ruxolitinib treatment in NB4 cells. Total cell extracts of NB4 cells treated with different doses of ruxolitinib for 6 h were submitted to immunobloting with anti-IRS2, anti-phosphotyrosine antibodies, and antibodies to detect downstream proteins. JAK2 was not constitutively phosphorylated in the NB4 cell line; the asterisk symbol (*) indicates HEL cells (included as a positive control). Membranes were reprobed with the antibody for detection of the respective total or phospho-protein, and developed with the ECL™ Western Blot Analysis System.

Supplementary Table S1: Primer sequences for *IRS2* and *HPRT*

Gene	Sequences
IRS2	FW: 5' GAGTGCACCCGTACCTATGGAA 3'<br>RW: 5' GAAATCCGGCTTTACCTTGAACT 3'
HPRT	FW: 5' GAACGTCTTGCTCGAGATGTGA 3'<br>RW: 5' TCCAGCAGGTCAGCAAAGAAT 3'

Supplementary Table S2: Primary antibodies used for Western blotting analysis

Antibody	Catalog number	Concentration
IRS2*,&	sc-1555	1:250
pTyr*	sc-508	1:1000
JAK2*,&	sc-294	1:1000
STAT3*	sc-7179	1:1000
STAT5*	sc-835	1:1000
Actin*	sc-1616	1:2000
P70S6K*	sc-8418	1:1000
p-P70S6K*	sc-7984	1:1000
AKT1/2/3*	sc-8312	1:2000
p-AKT*	sc-7985-R	1:500
p-JAK2#	3774S	1:1000
p-STAT3#	9131S	1:1000
p-STAT5#	9359S	1:1000
Caspase 3#	9665S	1:1000
p-ERK1/2§	44654G	1:1000
ERK1/2§	700012	1:2000

*Santa Cruz Biotechnology (Santa Cruz, CA, USA), #Cell Signaling Technology (Cell Signaling, Danvers, MA, USA), §Zymed (Invitrogen, Carlsbad, CA, USA); &Antibodies also used in confocal analysis.

Supplementary Table S3: Higher levels of *IRS2* mRNA expression in JAK2^{V617F} patients

Diagnosis	Essential thrombocythemia $n = 37$			Polycythemia vera $n = 30$			Primary myelofibrosis $n = 32$		
JAK2 mutation status	JAK2^{WT} $n = 22$	JAK2^{V617F} $n = 15$	p value	JAK2^{WT} $n = 2$	JAK2^{V617F} $n = 28$	p value	JAK2^{WT} $n = 11$	JAK2^{V617F} $n = 21$	p value
IRS2 expression*	0.31 (0.09–0.83)	0.50 (0.20–2.17)	.01	NA#	0.39 (0.00–5.65)	NA	0.12 (0.00–0.49)	0.52 (0.00–2.16)	.02

WT: wild-type; NA: not applicable
*Relative level of *IRS2* mRNA expression is indicated as median (minimum-maximum)
#The relative *IRS2* mRNA levels in JAK2^{WT} polycythemia vera patients were 0.01 for both patients ($n = 2$).
